# The deferred embryo transfer strategy improves cumulative pregnancy rates in endometriosis-related infertility: A retrospective matched cohort study

**DOI:** 10.1371/journal.pone.0194800

**Published:** 2018-04-09

**Authors:** Mathilde Bourdon, Pietro Santulli, Chloé Maignien, Vanessa Gayet, Khaled Pocate-Cheriet, Louis Marcellin, Charles Chapron

**Affiliations:** 1 Department of Gynecology Obstetrics II and Reproductive Medicine, Centre Hospitalier Universitaire (CHU) Cochin, Paris, France; 2 Department “Stress oxydant, prolifération cellulaire et inflammation”, Institut Cochin, INSERM U1016, Université Paris Descartes, Sorbonne Paris Cité, Paris, France; 3 Department “Development, Reproduction and Cancer”, Institut Cochin, INSERM U1016, Université Paris Descartes, Sorbonne Paris Cité, Paris, France; 4 Department of Histology-Embryology and Reproductive Biology, Centre Hospitalier Universitaire (CHU) Cochin, Paris, France; University of Crete, GREECE

## Abstract

**Background:**

Controlled ovarian stimulation in assisted reproduction technology (ART) may alters endometrial receptivity by an advancement of endometrial development. Recently, technical improvements in vitrification make deferred frozen-thawed embryo transfer (Def-ET) a feasible alternative to fresh embryo transfer (ET). In endometriosis-related infertility the eutopic endometrium is abnormal and its functional alterations are seen as likely to alter the quality of endometrial receptivity. One question in the endometriosis ART-management is to know whether Def-ET could restore optimal receptivity in endometriosis-affected women leading to increase in pregnancy rates.

**Objective:**

To compare cumulative ART-outcomes between fresh versus Def-ET in endometriosis-infertile women.

**Materials and methods:**

This matched cohort study compared def-ET strategy to fresh ET strategy between 01/10/2012 and 31/12/2014. One hundred and thirty-five endometriosis-affected women with a scheduled def-ET cycle and 424 endometriosis-affected women with a scheduled fresh ET cycle were eligible for matching. Matching criteria were: age, number of prior ART cycles, and endometriosis phenotype. Statistical analyses were conducted using univariable and multivariable logistic regression models.

**Results:**

135 in the fresh ET group and 135 in the def-ET group were included in the analysis. The cumulative clinical pregnancy rate was significantly increased in the def-ET group compared to the fresh ET group [58 (43%) vs. 40 (29.6%), p = 0.047]. The cumulative ongoing pregnancy rate was 34.8% (n = 47) and 17.8% (n = 24) respectively in the Def-ET and the fresh-ET groups (p = 0.005). After multivariable conditional logistic regression analysis, Def-ET was associated with a significant increase in the cumulative ongoing pregnancy rate as compared to fresh ET (OR = 1.76, CI95% 1.06–2.92, p = 0.028).

**Conclusion:**

Def-ET in endometriosis-affected women was associated with significantly higher cumulative ongoing pregnancy rates. Our preliminary results suggest that Def-ET for endometriosis-affected women is an attractive option that could increase their ART success rates. Future studies, with a randomized design, should be conducted to further confirm those results.

## Introduction

Endometriosis is a gynecologic disorder defined by the presence of endometrial-like tissue outside the uterine cavity [1]. Approximately 5–10% of reproductive-age women are affected by endometriosis, and at least one third of them are infertile [2]. While there is a fair amount of evidence demonstrating that endometriosis has a negative impact on fertility, the pathogenesis remains unclear. Current findings suggest several pathways may be involved, including inflammatory changes in peritoneal fluid resulting in altered sperm-oocyte interaction, reduced functional ovarian tissue, and hampered endometrial receptivity due to changes in the eutopic endometrium [2].

Differences in the eutopic endometrium between women with and without endometriosis is now well established [3–5]. Functional changes are believed to largely account for alteration in the quality of endometrial receptivity. Indeed, pro-inflammatory mediators, as well as candidate genes related to implantation failure and infertility, were found to be deregulated in endometrial tissues of women with endometriosis as compared to endometrial tissues from disease-free women [6,7].

Assisted reproductive technology (ART) is a reliable therapeutic option for managing endometriosis-related infertility [8]. ART protocols comprise a controlled ovarian stimulation (COS) to obtain oocytes for fertilization, which is usually followed by a fresh embryo transfer (ET). A growing concern in recent years is that COS, which generates a high level of sex-steroids, may alter endometrial receptivity by enhancing endometrial development, which could in turn contribute to a lower chance of becoming pregnant [9–11]. Technical improvements in vitrification have recently led to the emergence of a new strategy: the “freeze–all approach” [11,12]. This deferred frozen-thawed embryo transfer (Def-ET), initially developed to prevent ovarian hyper-stimulation syndrome in high-risk women [13], is now being applied to a wider population in order to improve endometrial implantation [14–17]. It consists of cryopreservation of the entire embryo-cohort, allowing the embryo transfer to be carried out separately from the COS in a subsequent cycle.

As endometriosis is an estrogen-dependent disease, it is reasonable to speculate that a high level of steroids induced by COS could interfere with endometrial receptivity: modification of gene expression profiles observed in women with endometriosis could be exacerbated by COS, leading to an inhospitable environment during the implantation process. Hence, COS could exacerbate the endometriosis-related reduction in the probability of implantation.

Thus, one of the main questions is whether deferring embryo transfers could restore optimal receptivity in endometriosis-affected women, thereby leading to an increase in pregnancy rates.

We hence undertook this matched cohort study to compare ART outcomes between fresh ET versus Def-ET in endometriosis-infertile women.

## Materials and methods

### Study design

We conducted a cohort study including all In Vitro Fertilization / Intra Cytoplasmic Sperm Injection (IVF/ICSI) cycles for endometriosis infertility between 01/10/2012 and 31/12/2014 in the ART unit at the university-based reproductive medicine center of our institution. The study cohort was matched in terms of patient age, the number of prior ART cycles (the number of prior ART cycle is defined as the number of prior COS leading to at least one embryo transfer with no pregnancy obtained [18]) and endometriosis phenotypes (superficial peritoneal endometriosis (SUP), ovarian endometrioma (OMA), or deeply infiltrating endometriosis (DIE) [19]. Two groups were compared: (i) a study group comprised of “exposed” women who received a Def-ET for the first transfer attempt and (ii) an “unexposed” control group comprised of women who received fresh ET for the first transfer attempt. For both groups, supernumerary embryos were frozen and transferred if pregnancy was not obtained first. All data were fully anonymized before use. Data collection and utilisation were approved by the National Data Protection Authority (Commission Nationale de l’Informatique et des Libertés, CNIL n° 1988293 v 0).

### Patient cohort and the matching procedure

For both groups the inclusion criteria for this cohort study were the following: women with endometriosis-related infertility, requirement of ART (IVF or ICSI), age ≤ 43 years, and having one or more embryo(s) available for transfer. Exclusion criteria were: women without endometriosis, vitrified oocyte procedures, no embryo obtained or transferred, and patients already included in another ART research protocol.

There are three phenotypes for endometriotic lesions: superficial peritoneal endometriosis (SUP), ovarian endometrioma (OMA), or deeply infiltrating endometriosis (DIE). In our referral center, all endometriosis infertile women underwent an appropriate pre-ART imaging work-up in order to obtain a clear diagnosis and staging of the endometriosis. For DIE and OMA phenotypes, diagnosis and staging of endometriosis was based on previously published imaging criteria using transvaginal ultrasound (TVUS) [20–22], magnetic resonance imaging (MRI) [23–26], or transrectal ultrasonography [27]. Additionally, for patients with a history of prior surgery, the diagnosis was also confirmed by histological proof of endometriosis. Patients were classified as SUP in the following cases: no OMA nor DIE lesions at the pre-ART imaging work-up and previously histologically proven superficial peritoneal endometriosis. Since these phenotypes are frequently associated, patients were assigned to the group corresponding to the most severe lesion according to a previous published classification [28,29], ordered from the least to the most severe, i.e. SUP, OMA, and then DIE.

The decision about whether to defer the embryo transfer or to perform a fresh embryo transfer for the first attempt was based on a joint decision by the patient and the doctor. The information process was conducted according to the specific elements of Braddock and colleagues [30] required for completeness of informed decision making. During the appointment, the practitioner fully involves the patient in the strategy to choose. After having informed the women of her role in decision making, the doctor clearly explains the nature of the decision and the 2 choices are proposed: The fresh embryo strategy or the deferred embryo strategy. Pros and cons of each strategy are carefully discussed during the appointment, especially the results of extended embryo culture, vitrification and survival rates. After assessment of the patient’s understanding, a joint decision by the patient and the doctor is made between the 2 strategies.

Matching criteria were the patients’ age, the number of prior ART cycles, and the endometriosis phenotypes (SUP, OMA, or DIE). Blind matching to the results was performed. Each Def-ET was matched to one fresh ET. Matching was performed by staff members who were cognizant of the matching criteria but who were otherwise blinded to the results. Matched records were used only once.

### Ovarian stimulation

Women were monitored and managed according to our institutional clinical protocols as reported previously [18]. Thus, all patients were synchronized using timed administration of an oral contraceptive (OC) containing 0.03 mg of ethynil estradiol (EE) and 0.15 mg of levonorgestrel (LNG) (Minidril, Pfizer Holding, Paris, France) [31]. Various COS protocols were used according to our institutional clinical protocols, with 150–450 IU/day of recombinant Follicle-stimulating hormone (FSH) (Puregon-MSD, Courbevoie, France; Gonal-F, Merck, Lyon, france) and urinary FSH (hMG, Menopur, Ferring Pharmaceuticals, Gentilly, France): (i) a GnRH antagonist protocol, (ii) a long agonist protocol, and (iii) a short agonist protocol [32]. Gonadotropin doses and the type of COS protocol were determined according to the individual patient’s characteristics. As previously described in the literature, antagonist protocol is preferentially used in the Def-ET strategy [12,33]. Final oocyte maturation was triggered when ≥ 3 ovarian follicles of ≥ 17 mm were visible by ultrasound: (i) for the Def-ET group, final oocyte maturation was achieved using either a single injection of 0.2 mg of GnRH agonist (Triptoreline, Decapeptyl, Ipsen, Boulogne Billancourt, France), or by 250 μg of recombinant hCG (rhCG, Ovitrelle, Serono, Lyon, France), according to the COS protocol; (ii) for the fresh ET group, final oocyte maturation was achieved using rhCG triggering. Oocyte retrieval was performed 35–36h later by transvaginal aspiration under ultrasound guidance.

### Oocyte insemination

Semen samples were collected by masturbation after a sexual abstinence period ranging from 2 to 5 days. Conventional IVF or ICSI were performed according to the sperm parameters [34]. Fertilization was assessed by the presence of two pronuclei (2PN) and two polar bodies at 17-18h following oocyte insemination or injection.

### Embryo culture, cryopreservations and thawing

Concerning prolonged cultures, embryos were transferred into a 50 μl droplet of one-step Global culture medium (LifeGlobal, USA) and cultured until day 5 or 6 at 37°C in an atmosphere of 5% CO_2_, 5% O_2_ and 90% N_2_. The culture medium was changed on day 3. Embryo morphology was evaluated on the morning of day 5 and 6. Blastocysts were scored according to the grading system of Gardner and Schoolcraft [35] and considered eligible for cryopreservation on day 5 or 6 if qualifying as full (B3) or expanded (B4-5) blastocysts with a type A-C inner cell mass and/or a type A-C trophectoderm. Blastocyst that did not meet these criteria on day 5 were kept in culture and re-examined on day 6. Blastocysts with a type “C” inner cell mass (ICM) and a type “C” trophectoderm were not cryopreserved regardless of their degree of expansion and the day of observation (days 5–6).

The precise vitrification and thawing protocol in our unit has been reported in detail previously [18]. Briefly, embryo vitrification was performed using closed Cryo-Bio-System vitrification (CBS-VIT) High Security (HS) straws in combination with DMSO-EG-S as the cryoprotectants (Irvine Scientific Freeze Kit). For thawing, the Irvine Scientific Thaw Kit was used. Zygotes were warmed the day prior to the embryo transfer and kept in culture for 24 hours in the same culture medium (50 μl droplet of one-step Global culture medium (LifeGlobal, USA) at 37°C in an atmosphere of 5% CO_2_, 5% O_2_, and 90% N_2_). On day 2, the embryos were morphologically assessed according to the criteria published in the Istanbul Consensus workshop on embryo assessment guide [36]. One or two best quality embryos were chosen for transfer. Supernumerary embryos, if any, were maintained in extended culture, and vitrified if they reached the blastocyst stage. Blastocysts were warmed on the day of transfer. When the warmed blastocyst had < 50% intact cells, an additional blastocyst was warmed if available. If the blastocyst was > 50% intact, expansion and re-expansion were assessed 2–3 hours later.

### Endometrial preparation and embryo transfer

In the fresh ET group, all of the women began progesterone treatment (200 mg vaginal capsule *t*.*i*.*d*, Utrogestan, Besins International, Montrouge, France) the day of the oocyte retrieval, and estradiol (E2) was delivered transdermally (0.2 mg/day, through two Vivelledot 100 systems simultaneously, Novartis Pharma SA, Rueil malmaison, France) or orally (8 mg/day, Provames, Sanofi Aventis, Paris, France) 48h after the ET. Day-2 embryos were assessed morphologically, and the one or two embryos deemed to be the most suitable were selected for transfer. In the Def-ET group, all of the women received progesterone (one 200 mg vaginal capsule, daily) for 10 days in order to assure proper occurrence of menses. Embryo transfers were scheduled 4–5 weeks later. For this, women received an estradiol (E2) priming regimen that was delivered transdermally (0.2 mg/day) or orally (8 mg/day). Patients were examined again 2–3 weeks after menses in order to assess endometrial thickness and to determine progesterone levels. When conditions were appropriate (e.g. endometrium thickness ≥ 7mm and progesterone < 1.5 ng/ml), vaginal progesterone treatment was initiated at a dose of 200 mg *t*.*i*.*d*. Day-2 cleaved-stage embryos were transferred on the 4^th^ day of progesterone exposure. Blastocysts were transferred immediately on the 5^th^ day of progesterone exposure. The best quality embryos were chosen for transfer. The women who became pregnant by these procedures continued with the same dose of progesterone and E2 treatment until 12 weeks of gestation.

### Data analysis and statistics

The general characteristics of the patients in both groups were recorded prospectively, prior to the COS. The following data were collected: age (in years); height (in meters); weight (in kilograms), body mass index (BMI, calculated as weight (kg)/[height (m)] ^2^); the number of prior ART cycles; the duration of their infertility; ovarian reserve (day 3 FSH, Luteinizing hormone (LH), estradiol; antral follicle count (AFC)); AMH levels; associated factors of infertility (e.g. a male factor, tubal factor, and associated adenomyosis). Associated adenomyosis was diagnosed using imaging criteria based on TVUS and MRI [37].

Clinical pregnancy rates (cPR) were determined by ultrasonographic documentation of at least one fetus with a heart beat at 6–7 weeks of gestation [38]. The ongoing pregnancy rate (oPR) was defined as the sonographic detection of one or more intrauterine fetuses with a positive heartbeat at 12 weeks of gestation [39]. Live birth rate (LBR) was defined as delivery of any viable infant at 22 weeks or more of gestation [38].

Cumulative cPR, oPR and LBR were the proportion of women that had at least one clinical pregnancy, ongoing pregnancy and live birth, respectively, whether from the first transfer attempt or subsequent transfers of frozen–thawed supernumerary embryos [15] after the oocyte retrieval. Once a woman obtained a pregnancy from IVF/ICSI she did not contribute any more to the cumulative rates [40].

The main ART outcome measure was cumulative cumulative oPR. Secondary outcomes were Cumulative cPR, cumulative LBR and cPR, oPR and LBR after the first ET. All of the data were entered into a digital database and analyzed using SPSS software (SPSS Inc., Chicago, IL, USA). A *p* value < 0.05 was considered to be statistically significant. Given the matched design of our groups (fresh and Def-ET) we used matched-pair statistics for the univariate statistical analysis. Dichotomous variables were compared by the McNemar test, and continuous variables were assessed by paired *t*-test, as appropriate. For subgroup analysis of non-matched groups we used the unpaired *t*-test for continuous variables, and the Pearson’s χ^2^ test or the Fisher’s exact test for qualitative variables. Then, a second analysis was performed to identify risk factors of cumulative ongoing pregnancy with a conditional logistic regression analysis. Potential confounding factors found to be statistically significant at the threshold of *p* ≤ 0.10 after an univariable analysis and variables with clinical relevance were included in the conditional multiple logistic regression model.

The model built to retain variables into the final model was processed according to scientific knowledge and clinical relevance of variables.

## Results

### Study population

**Fig 1:**
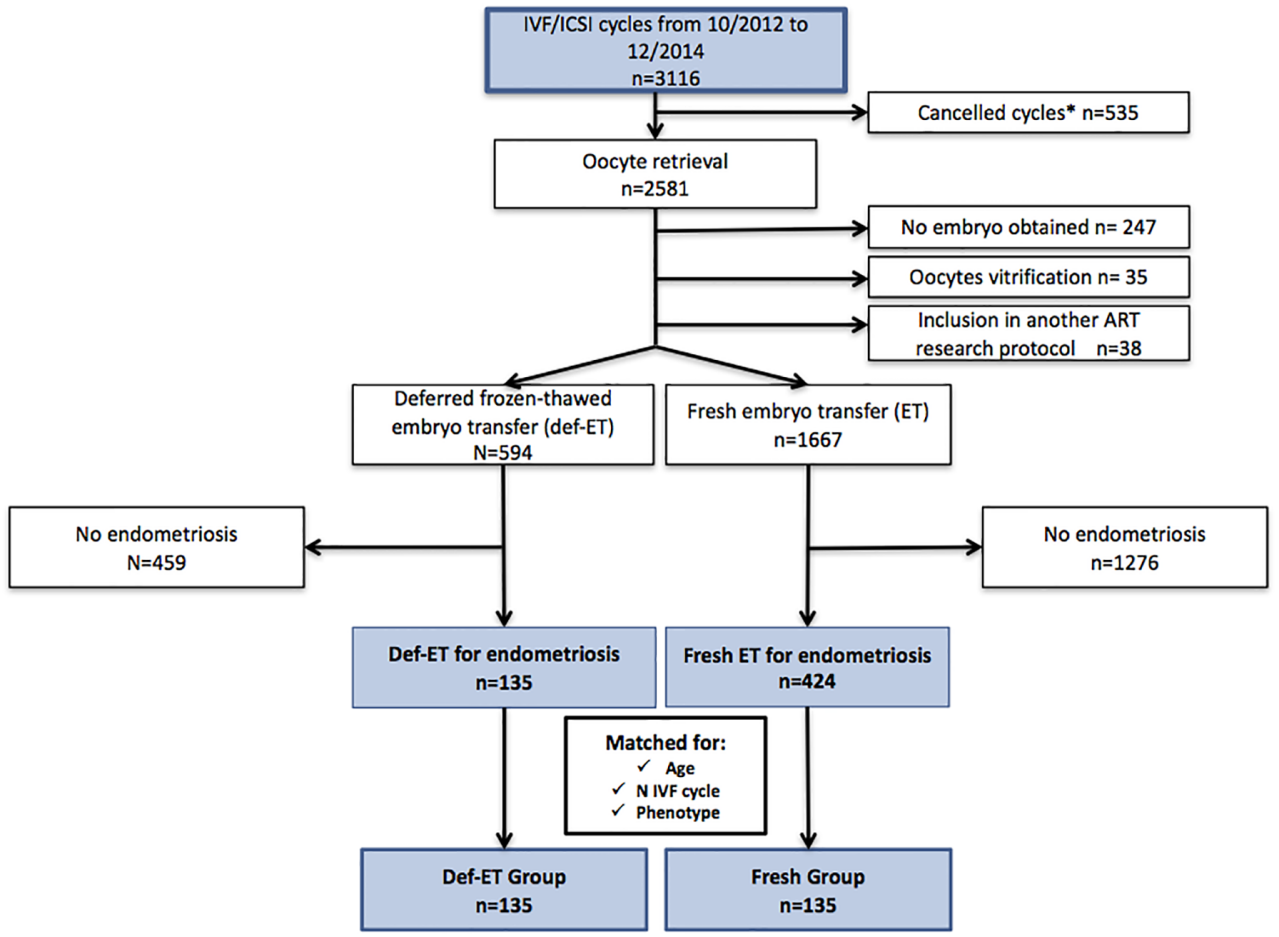
Patient inclusion flowchart. IVF/ICSI, in vitro fertilization / intra cytoplasmic sperm injection; Def-ET: Deferred frozen-thawed embryo transfer—*Cancelled cycles: Poor response—Personal or medical (e.g. non-gynecological) reasons.

The process of our cohort selection is detailed in Fig 1. From the initial cohort of 3 116 ART procedures scheduled in our reproductive medicine unit between the 1^st^ of October 2012 and the end of December 2014, 535 cycles were cancelled before oocyte retrieval. Three hundred and twenty were excluded from the study for the following reasons: no embryo obtained (n = 247), oocyte vitrification procedure (n = 35), and inclusion in another ART research protocol (n = 38). For the remaining 2 261 cycles, we excluded women without endometriosis [459 women who received a Def-ET and 1 276 women who received a fresh ET] from the study. Overall, 135 Def-ET cycles and 424 fresh ET cycles performed for endometriotic women were eligible for matching. Of these, 135 pairs of fresh and deferred cycles were matched for age, ART ranking, and the endometriosis phenotype. The endometriosis phenotypes were as follows: SUP, 27 patients (10.00%); OMA, 66 patients (24.44%); and DIE, 177 patients (65.56%). One hundred and two (37.78%) DIE patients exhibited an associated OMA.

The patient characteristics are presented in Table 1. A prior history of surgery for endometriosis was significantly more frequent in the fresh ET group [100 (74.07%) versus 43 (31.85%), p < 0.001]. Moreover, the distribution of the type of stimulation protocol was different between the two groups. For the fresh group, the long agonist protocol [67 (49.63%) versus 16 (11.85%) for the Def-ET group] and the short agonist protocol [41 (30.37%) versus 9 (6.67%) for the Def-ET group] were more often performed than in the Def-ET group. Therefore, the total dose of injected gonadotropin was significantly higher for the fresh group (3 064.1 ± 1 210.3 versus 2 498.5 ± 809.9, p < 0.001). Conversely, age, the number of prior ART procedures, BMI, the duration of the infertility, gravidity and parity, associated infertility factors, ovarian reserve parameters, and the endometriosis phenotypes, were not significantly different between the two groups (Table 1 and S1 Table).

**Table 1:** Baseline characteristics in matched fresh and deferred frozen-thawed embryo transfer groups.

	Fresh-ET group (n = 135)	Def-ET group (n = 135)
**Patient’s Age at COS (years)** (mean ± SD)	34.3 ± 3.9	34.3 ± 4.1^a^
**Number of prior ART cycles** (mean ± SD)	1.9 ± 1.0	2.0 ± 1.1^a^
**Duration of the infertility (years)** (mean ± SD)	4.4 ± 2.3	4.7 ± 2.7
**BMI (kg/m2)** (mean ± SD)	22.3 ± 3.3	22.8 ± 3.6
**Gravidity—**n (%)		
**0**	101 (74.8)	112 (83.0)
**≥1**	34 (25.2)	23 (17.0)
**Parity—**n (%))		
**0**	123 (91.1)	125 (92.6)
**≥1**	12 (8.9)	10 (7.4)
**Associated male infertility—**n (%)	26 (19.3)	19 (14.1)
**Associated tubal factor—**n (%)	12 (8.9)	12 (8.9)
**Associated adenomyosis—**n (%)	56 (41.5)	57 (42.2)
**Prior endometriosis surgery—**n (%)	100 (74.07)	43 (31.85)
**Patient’s ovarian reserve—**n (%)		
AMH ≤ 1.5ng/ml	34 (25.19)	23 (17.04)
AFC ≤ 10	63 (46.67)	49 (36.30)
**Endometriosis phenotypes** n (%)		
SUP	12 (8.89)	15 (11.11)^a^
OMA	34 (25.19)	32 (23.70)
DIE	89 (65.93)	88 (65.19)
**Stimulation protocol** n (%)		
Long agonist protocol	67 (49.63)	16 (11.85)^b^
Antagonist Protocol	27 (20.00)	110 (81.48)^b^
Short agonist protocol	41 (30.37)	9 (6.67)^b^
**Total dose of injected gonadotropin (IU)** (mean ± SD)	3064.1 ± 1210.3	2498.5 ± 809.9^c^

Fresh-ET, Fresh embryo transfer; Def-ET, Deferred embryo transfer; ART, Assisted reproductive technology; COS, controlled ovarian stimulation; BMI, Body mass index; FSH, Follicle-stimulating hormone; LH, Luteinizing hormone; AFC, Antral follicle count; AMH, Anti mullerian hormone; SUP, superficial peritoneal endometriosis; OMA, endometriomas; DIE, Deep infiltrating endometriosis; GnRH, Gonadotropin-releasing hormone

^a^ p value not provided because groups were matched for age, number of prior ART cycles and endometriosis phenotypes

^b^ Same superscript letter in a row indicate a statistically significant difference (P < 0.05) using a McNemar test.

^c^ Same superscript letter in a row indicate a statistically significant difference (P < 0.05) using a paired t test.

### IVF-ICSI outcomes

Table 2 depicts IVF/ICSI outcomes in matched fresh and Def-ET groups after univariable analysis. This non-adjusted analysis found a lower number of oocytes retrieved in the fresh group (7.4 ± 4.3 versus 9.9 ± 7.0 in the Def-ET group, p < 0.001) (Table 2) and the cumulative cPR [40 (29.6%) versus 58 (43.0%), p = 0.047], the cumulative oPR [24 (17.8%) vs. 47 (34.8%), p = 0.005] and the cumulative LBR [21 (15.6%) vs. 41 (29.6%), p = 0.012] were significantly higher in Def-ET group as compared to fresh ET group. Conversely, the miscarriage rate was significantly higher in the fresh ET group. The multiple pregnancy rates were not significantly different between the two groups (Table 2).

**Table 2:** IVF/ICSI-characteristics and outcomes in matched fresh and deferred frozen embryo transfer groups.

	Fresh-ET group (n = 135)	Def-ET group (n = 135)	p-value
**Number of oocytes retrieved** (mean ± SD)	7.4 ± 4.3	9.9 ± 7.0	*0*.*001* ^pt^
**Total number of embryos transferred**	278	224	*NA*
**Mean No. of embryos transferred** (mean ± SD)	2.1 ± 0.9	1.7 ± 0.9	*<0*.*001* ^pt^
**Total number of embryo transfers**	155	170	*NA*
**Mean No. of transfers** (mean ± SD)	1.2 ± 0.4	1.3 ± 0.7	*0*.*132* ^pt^
**Cumulative clinical pregnancy rate—**(n,%)	40 (29.6)	58 (43.0)	*0*.*047* ^mn^
Miscarriage—(n,%)	16/40 (40.0)	11/58 (19.0)	*0*.*022* ^k^
Multiple pregnancy—(n,%)	7/40 (17.5)	5/58 (8.6)	*0*.*220* ^k^
**Cumulative ongoing pregnancy—**(n,%)	24 (17.8)	47 (34.8)	*0*.*005* ^mn^
**Cumulative live birth rate** ^a^—(n,%)	21 (15.6)	41 (29.6)	*0*.*012* ^mn^

IVF/ICSI, in vitro fertilization / intra cytoplasmic sperm injection; Fresh-ET, Fresh embryo transfer; Def-ET, Deferred frozen- thawed embryo transfer; NA, non applicable

^*pt*^, Paired t-test;

^*mn*^, McNemar test;

^*k*^ Pearson’s chi-square test.

^**a**^ 2 and 5 women were lost to follow up in Fresh and Def-ET group respectively

IVF-ICSI outcomes after the first-ET were depicted in S2 Table. Ongoing PR [42 (31.1%) versus 24 (17.8%), p = 0.003] and LBR [39 (28.9%) versus 21 (15.6%), p = 0.025] after the first-ET were significantly higher in Def-ET group as compared to fresh ET group (S2 Table). The miscarriage rate was significantly higher in the fresh ET group.

### Risk factors for cumulative ongoing pregnancy

Univariable analysis comparing patients who became pregnant and those who did not is presented in Table 3. A prior history of surgery for endometriosis (OR = 0.35; 95% CI = 0.16–0.78; p = 0.010) and having a Def-ET versus a Fresh ET (OR = 1.94; 95% CI = 1.19–3.18; p = 0.008) were statistically significantly associated with cumulative ongoing pregnancy.

**Table 3:** Risk factors of cumulative ongoing pregnancy: Results from the conditional univariable and multivariable logistic regression analysis.

Parameters	No cumulative ongoing pregnancy (-) (n = 199)	Cumulative ongoing pregnancy (+) (n = 71)	OR (95% CI)	p value	Adjusted OR(95%CI)	p value
**Duration of the infertility (years) (mean ± SD)**	4.7 ± 2.6	4.0 ± 2.1	0.90 (0.77–1.06)	0.199		
**BMI (kg/m2) (mean ± SD)**	22.7 ± 3.4	22.2 ± 3.5	0.97 (0.89–1.06)	0.482		
**Gravidity—n (%)**				0.488		
0	157 (78.9)	56 (78.9)	1			
≥1	42 (21.1)	15 (21.1)	1.34 (0.59–3.06)			
**Parity—n (%)**				0.711		
0	184 (92.5)	64 (90.1)	1			
≥1	15 (7.5)	7 (9.9)	1.23 (0.41–3.68)			
**Associated male infertility—n (%)**	37 (18.6)	8 (11.3)	0.48(0.21–1.12)	0.089	0.59 (0.27–1.32)	0.439
**Associated tubal factor—n (%)**	20 (10.1)	4 (5.6)	0.67 (0.19–2.36)	0.530		
**Associated adenomyosis—n (%)**	88 (44.2)	25 (35.2)	0.75 (0.36–1.54)	0.430		
**Prior endometriosis surgery—n (%)**	116 (58.3)	27 (38.0)	0.35 (0.16–0.78)	0.010	0.66(0.39–1.12)	0.121
**Patient’s ovarian reserve—n (%)**						
AMH ≤ 1.5ng/ml	46 (23.1)	11 (15.5)	0.44(0.19–1.02)	0.056	0.67(0.32–1.45)	0.313
AFC^a^ ≤10	87 (43.7)	25 (35.2)	0.56(0.30–1.06)	0.075		
**Stimulation protocol—n (%)**						
GnRH antagonist	66 (33.2)	17 (23.9)	1		1	
Long agonist protocol	90 (45.2)	47 (66.2)	0.62(0.30–1.29)	0.203	1.32(0.54–3.23)	0.541
Short agonist protocol	43 (21.6)	7 (9.9)	0.60 (0.23–1.57)	0.298	0.95 (0.32–2.85)	0.927
**Total dose of injected gonadotropin (IU) (mean ± SD)**	2884.2 ± 1088.7	2492.4 ± 949.4	1.00(0.99–1.01)	0.136	1.00(0.99–1.01)	0.439
**Type of embryo transfer—n (%)**				0.008		0.028
Fresh-ET	111 (55.8)	24 (33.8)	1		1	
Def-ET	88 (44.2)	47 (66.2)	1.94 (1.19–3.18)		1.76(1.06–2.92)	
**Number of oocytes retrieved (mean ± SD)**	8.06 ± 5.3	10. ± 7.1	1.06 (0.99–1.12)	0.065	1.04 (1.01–1.07)	0.038

BMI, Body mass index; AMH, Anti-mullerian hormone; AFC, Antral follicle count; GnRH, Gonadotropin-releasing hormone; Fresh-ET, Fresh embryo transfer; Def-ET, Deferred embryo transfer; n number of cases, OR Odds ratio, CI Confidence interval;

^a^ omitted from multivariable regression model due to multicollinearity

A multivariable analysis was performed to identify risk factors of cumulative ongoing pregnancy. Potential confounding factors included in the model were: An associated male infertility, a prior history of surgery for endometriosis, AMH level (≤ 1.5ng/ml versus >1.5 ng/ml), the type of stimulation protocol, the total dose of the injected gonadotropin (IU), the type of ET (fresh versus Def-ET), and the number of oocytes retrieved. The type of ET (fresh versus Def-ET) (fresh embryo transfer, OR = 1, Def-ET, OR = 1.76; 95% CI = 1.06–2.92; p = 0.028) and the number of oocytes retrieved (OR = 1.04; 95% CI = 1.01–1.07; p = 0.038) remained independent factors associated with cumulative ongoing pregnancy, as shown in Table 3.

## Discussion

### Main findings

This study compared ART outcomes of endometriosis-affected women, according to the type of ET: fresh versus deferred. In our population, a significantly higher cumulative pregnancy rate was observed after a deferred ET strategy. Based on a multivariable multivariate analysis, there were two independent predictive factors for ongoing pregnancy rates after ART: the number of oocytes retrieved and the type of ET (deferred ET strategy), both of which enhanced pregnancy rates.

### Strengths and limitations

1) Our study focused on the important issue of management of endometriosis-related infertility. To the best of our knowledge, this is the first study to specifically examine ART outcomes in endometriosis-affected women after a fresh versus a deferred ET strategy; 2) Given the disease heterogeneity, we selected patients with well-defined endometriosis phenotypes (SUP, OMA, or DIE) [41]. For DIE and OMA phenotypes, we only included patients whose diagnosis of endometriosis was based on stringent image-based criteria [20–22,25]; Additionally, for patients with a history of prior surgery for endometriosis, the diagnosis was also confirmed by histological proof of endometriosis. For the SUP phenotype, the diagnosis was proven histologically, and no OMA nor DIE lesions at the pre-ART imaging work-up were identified; 3) The strengths of the study are also derived from some of the specifics of the methodological design: this is a matched controlled study including a large number of endometriosis affected-patients (270 women undergoing IVF/ICSI cycles) and great care was taken to minimize the possibility of biases. As advanced age and prior ART failure could have a negative influence on ART outcomes [42,43] we selected the age of the women and the number of prior ART as matching criteria. Additionally, we decided to match based on the endometriosis phenotypes. Since the link between endometriosis phenotypes and ART outcomes is a matter of debate [8,44], it seemed relevant to have comparable groups in terms of endometriosis phenotypes. 4) In order to compare the fresh versus the deferred ET strategy, in this study we used the cumulative pregnancy rate per oocyte retrieval, as published previously [40,45]. By providing an all-inclusive success rate, this analysis is of considerable relevance for clinicians since cryopreservation has become an integral part of ART. 5) Lastly, numerous epidemiological variables were collected prospectively through face-to-face interviews before ART (in regard to surgical history, infertility data, and ovarian stimulation characteristics).

Despite the precautions taken, our study may still be subject to certain shortcomings and/or biases. 1) This study was conducted in a referral center for endometriosis management. The women referred to our center may therefore have suffered from particularly severe forms of endometriosis (65.6% of the women in our study had a DIE phenotype). This referral bias for women with severe lesions might have influenced the ART outcomes. However, in light of the criteria for matching, the distribution of endometriosis phenotypes was identical in both groups; 2) Some differences persisted between the groups: The women in the fresh ET group had significantly more often a prior history of surgery for endometriosis, and this could potentially have an impact on the ovarian response to stimulation [46]. We are cognizant that women in the fresh group had a less efficient response to stimulation. Despite a higher mean dose of injected gonadotropins for the fresh group, the mean number of oocytes retrieved was lower compared to the deferred ET group.

In order to evaluate the possible impact of these differences on pregnancy outcomes and therefore to determine which factors are independently linked to cumulative ongoing PR, we have performed a second analysis comparing patients who became pregnant to those who did not in our study population. After a multivariable logistic regression analysis, two factors were independently associated with cumulative ongoing PR: the type of transfer (i.e. the fresh versus deferred ET strategies) and the number of oocytes retrieved. Having ‘a prior history of surgery for endometriosis’ was not found to be independently associated with cumulative ongoing pregnancy rates. 3)Finally, this analysis consists in a preliminary cohort study. We are aware that randomized controlled studies should confirmed those results. Bias inherent in the study design cannot be excluded. However, it still allows us to identify the deferred embryo strategy as a new approach for endometriosis infertile women.

### Interpretation

The goal of this preliminary study was to compare the overall strategy of embryo transfer (i.e. fresh versus deferred). Given the existence of differences in regard to the number of oocytes, these results need to be interpreted with caution. We were nonetheless able to show that the deferred ET procedure could constitute a promising strategy for patients with endometriosis-related infertility. Indeed, in our model, carrying out a deferred ET rather a fresh ET was significantly linked with becoming pregnant. Our study hence provides new insight regarding ART management in endometriosis-related infertility. Deferring the ET relative to the ovarian stimulation appears to improve cumulative pregnancy rates in endometriosis-affected women undergoing IVF/ICSI.

Based on what has previously been reported the literature, it appears that endometrial alterations occur in women with endometriosis. Endometriosis induces changes in the eutopic endometrium in a natural cycle [3,4]. These endometrial-alterations have been related to the occurrence of a self-survival loop involving different pathways: inflammation, angiogenesis, and a disruption of steroidogenesis [47]. The endometrium in endometriosis-affected women could be less receptive than in women without endometriosis [2]. Even if there is currently no formal evidence for this, ovarian stimulation is widely thought to be a factor that adversely affects endometrial receptivity [11]. Our results are in accordance with the hypothesis that endometrial receptivity in endometriosis-affected women could be aggravated by ovarian stimulation.

The high rate of miscarriages (27.55%) that occurred in our study is consistent with findings in previous reports that investigated the association between endometriosis and miscarriages. As published previously, the risk of pregnancy loss in endometriosis-affected women is higher than in women without endometriosis [48–50]. Additionally, in the present study, a significantly higher rate of pregnancy loss was observed after a fresh versus a def-ET strategy (p = 0.022). These results are in accordance with a previous randomized controlled study focusing on ART outcomes in case of polycystic ovary syndrome after a fresh or a deferred ET [17].

The best practice for treating moderate to severe endometriosis-related infertility is still a matter of debate in the medical community. Nevertheless, patients with endometriosis-associated infertility exhibit good pregnancy rates after IVF/ICSI treatment compared to women with no associated endometriosis [51,52]. Some gynecologists are concerned that ART could negatively impact on endometriosis related symptoms. This concern no longer appears to be justified since recent prospective studies have shown no worsening of pain scores during IVF/ICSI procedures in endometriosis-affected women as compared to women without endometriosis [53,54]. Furthermore, the use of GnRHa triggering in association with a def-ET strategy has been recently been reported to limit pain symptoms during IVF/ICSI treatment [55]. Therefore, the def-ET strategy could be beneficial for endometriosis-affected women undergoing IVF/ICSI: firstly, by increasing cumulative pregnancy rates and, secondly, in association with a GnRHa triggering, by limiting physically painful symptoms [55].

## Conclusion

In conclusion, these preliminary results suggest that deferred ET for endometriosis-affected women is an attractive option to increase ART success rates. Furthers studies, with a randomized design, should be conducted to more firmly confirm whether def-ET enhances pregnancy rates in endometriosis-affected women and to confirm these preliminary results.

## Supporting information

S1 Table Database. (XLSX)

S2 Table IVF/ICSI-characteristics and outcomes in matched fresh and deferred frozen embryo transfer groups after the first embryo transfer. (DOCX)
