# Supplementary material for: The deferred embryo transfer strategy improves cumulative pregnancy rates in endometriosis-related infertility: A retrospective matched cohort study
Source: PLoS One. 2018 Apr 9;13(4):e0194800. doi: 10.1371/journal.pone.0194800 (PMC5890985; doi:10.1371/journal.pone.0194800)
Supplement: S2 Table — (DOCX) [file pone.0194800.s002.docx]

|  | Fresh-ET group  (n=135) | Def-ET group  (n=135) | p-value |
| --- | --- | --- | --- |
| **Total number of embryos transferred** | 243 | 186 | *NA* |
| **Mean No. of embryos transferred** (mean ± SD) | 1.8 ± 0.4 | 1.4 ± 0.5 | *<0.001 ^pt^* |
| **Implantation rate** (mean ± SD) | 0.2 ± 0.3 | 0.3 ± 0.4 | *0.008 ^pt^* |
| **Clinical pregnancy rate -** (n,%) | 39 (28.9) | 50 (37.0) | *0.235 ^mn^* |
| Miscarriage - (n,%) | 15(38.5) | 8(16.0) | *0.016 ^k^* |
| **Ongoing pregnancy rate -** (n,%) | 24 (17.8) | 42 (31.1) | *0.003 ^mn^* |
| **Live birth rate^a^ -** (n,%) | 21 (15.6) | 39 (28.9) | *0.025 ^mn^* |

**S2 Table. IVF/ICSI-characteristics and outcomes in matched fresh and deferred frozen embryo transfer groups after the first embryo transfer**

IVF/ICSI, in vitro fertilization / intra cytoplasmic sperm injection; Fresh-ET, Fresh embryo transfer; Def-ET, Deferred frozen- thawed embryo transfer; NA, non applicable

*^pt^*, Paired t-test; *^Mn^*, McNemar test; *^k^* Pearson’s chi-square test.

**^a^** 2 and 5 women were lost to follow up in Fresh and Def-ET group respectively
